# Evaluation of the occurrence of multiple paternity in *Squalus acanthias* in the South Atlantic region using nuclear markers

**DOI:** 10.1590/1678-4685-GMB-2026-0013

**Published:** 2026-07-03

**Authors:** Beatriz Rochitti Boza, Yan Torres, Gabriel Monteiro de Lima, Sergio Matías Delpiani, Gabriela Delpiani, Fausto Foresti, Claudio Oliveira, Vanessa Paes da Cruz

**Affiliations:** 1 Universidade Estadual Paulista “Júlio de Mesquita Filho” (UNESP), Botucatu, SP, Brazil .; 2 Universidade Estadual Vale do Acaraú, Sobral, CE, Brazil.; 3 Universidade Federal do Pará (UFPA), Bragança, PA, Brazil.; 4 Instituto de Investigaciones Marinas y Costeras, Mar del Plata, Departamento de Biología, Facultad de Ciencias Exactas y Naturales, Grupo de Biotaxonomía Morfológica y Molecular de Peces, Buenos Aires, Argentina.; 5 Consejo Nacional de Investigaciones Científicas y Técnicas (CONICET), Buenos Aires, Argentina.

**Keywords:** ddRAD, genomic, shark, SNPs, kinship

## Abstract

Understanding shark reproductive modes is crucial for conservation, as these K-strategist species are vulnerable to overexploitation. The spiny dogfish (*Squalus acanthias*), a small shark listed as ‘vulnerable’ by the IUCN, has a 22-month gestation period and a reproductive output ranging from 1 to 21 pups per litter. This study aimed to investigate multiple paternity in *S. acanthias* using Single Nucleotide Polymorphism (SNP) markers. Samples from six litters, comprising 40 individuals collected in Argentina, were analyzed using a ddRADseq library. SNP markers were screened with the STACKS pipeline, and kinship and paternity were analyzed using COANCESTRY and COLONY software. Results revealed 1,021 to 1,620 SNPs per litter, with multiple paternity detected in all litters. The number of sires per litter ranged from 2 to 4. No correlation was found between litter size and multiple paternity, suggesting this behavior may enhance genetic diversity. The species’ size and sex segregation, coupled with females in shallower waters, increase their vulnerability to fishing pressure. Overfishing and bycatch exacerbate the reduction in sexually mature individuals, threatening population recovery. This study highlights the need for management policies that incorporate reproductive strategies, especially for species like *S. acanthias* with complex life histories and low recovery rates.

## Introduction

Knowledge of species’ mating systems is fundamental for understanding species behavior and helpul for developing strategies for their conservation (Pratt and Carrier, 2001; Lamarca et al., 2020 a ). Polyandry involves multiple males fertilizing a single female within the same reproductive season, a phenomenon known as multiple paternity (Daly-Engel et al., 2006; Karl, 2008), observed across various taxa including all vertebrate classes (reviewed in Taylor et al., 2014). This behavior is common among species with internal fertilization (Birkhead, 2000; DeWoody and Avise, 2001).

**Figure 1 -:**
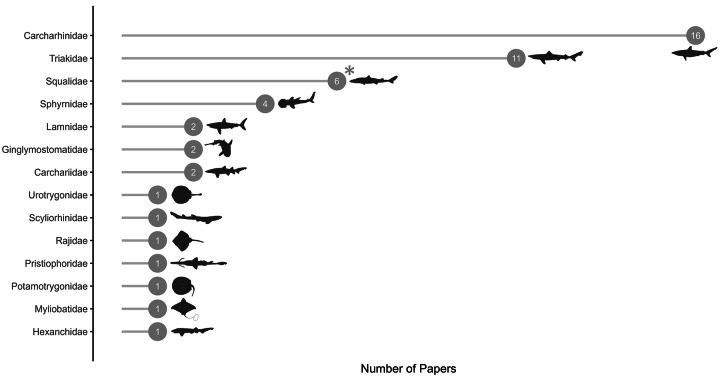
Number of studies reporting multiple paternity in elasmobranch families, representing 14 families and 33 species of sharks and rays. The numbers inside the circles indicate the number of published studies for each family. The asterisk (*) in Squalidae indicates that the present study is included in the count.

Eight orders of elasmobranchs, including six orders of sharks and two orders of rays, have shown evidence of multiple paternity: Carcharhiniformes, Hexanchiformes, Lamniformes, Orectolobiformes, Squaliformes, Pristiophoriformes, Rajiformes, and Myliobatiformes (Figure 1) (see Table S1). Sharks have evolved various reproductive modes over their evolutionary history, including oviparity (egg-laying) and different forms of viviparity (internal fertilization) (Chapman et al., 2004). Despite these adaptations, little is known about sperm storage and competition in most shark species, although females of many shark species can store sperm in the oviducal gland for extended periods, ranging from months to years before fertilization (Pratt, 1993; Hamlett et al., 1998, 1999; Lamarca et al., 2024).

The reasons for this behavior are still a knowledge gap to be filled in studies of cartilaginous fish (Lamarca et al., 2020a). Fox et al. (2019) proposed two probable ideas for the occurrence of polyandry in animals, the first consists of genetic benefits, since parentage of the offspring tends to be observed in males that have the aptitude above-average genetic fitness, and the second is a possible imbalance between mating rates for males and females, which can lead to sexual conflicts.

More specifically in elasmobranchs, multiple matings have obvious benefits for male fitness, as they are likely to generate more offspring with each additional mating. For females, the benefits of multiple matings can only be considered genetic (Lyons et al., 2017), as sharks do not form monogamous couples, do not provide parental care for the offspring, and do not share resources after mating (Pratt and Carrier, 2001). The description of elasmobranch mating systems is fundamental for the design and implementation of conservation and management strategies (Pratt and Carrier, 2001), particularly for those sustaining economically important fisheries or under vulnerable conservation status.

In addition, males are aggressive during mating attempts, whereas females often suffer serious injuries during copulation, making them more susceptible to predation during and after mating attempts, as well as more exposed to the risks of blood disorders, infections, and sexually transmitted diseases (Daly-Engel et al., 2010; Byrne and Avise, 2012; Ritter and Amin, 2019). Thus, multiple mating for females may be a means by which they avoid excessive harassment by males, where the cost of resisting to mating is greater than the cost of accepting mating, a hypothesis known as “convenience polyandry” (Portnoy et al., 2007; DiBattista et al., 2008).

*Squalus acanthias* Linnaeus, 1758, popularly known as “spiny dogfish”, is considered a small shark (~1.5m total length), is highly migratory and with a worldwide distribution, except in the tropical and polar regions (Compagno, 1984). The spiny dogfish is a mesopredator species with a highly diversified diet, including small invertebrates, teleost fishes, crustaceans, and cephalopods (Dunn et al., 2013). This species can live up to 75 years (Cailliet et al., 2001) and sexual maturation occurs between 60-70 cm TL (total length) for males and 75-90cm TL for females (Muus and Nielsen 1999), accompanied by long gestational periods (18 to 24 months) with an average of 1 to 21 pups per gestation (Compagno, 1984).

Like many elasmobranchs, *S. acanthias* has K-strategist characteristics, with slow growth rates, low fecundity, and late sexual maturation (Compagno, 1984; King and McFarlane, 2003; Dell’Apa et al., 2015), consequently, it is sensitive to overfishing (Gračan et al., 2020; Haque et al., 2021). Therefore, the complete recovery of the stock could take approximately ten years, which is approximately the period required to reach female sexual maturity (7.5 years) plus gestation time (two years) (Bargione et al., 2019).

Globally, *S. acanthias* is classified as VU “Vulnerable” by the International Union for Conservation of Nature (IUCN) red list of endangered species (Finucci *et al.,* 2020). Despite its threatened conservation status, the spiny dogfish is an economically important species, used for food consumption, obtaining liver oil, vitamins, sandpaper, leather, and fertilizers (Compagno, 1984). Moreover, the consumption of *S. acanthias* meat can be harmful to human health due to the high accumulation of heavy metals, as reported by the study by Kirov et al. (2022).

Genetic studies on *S. acanthias* mostly use microsatellite markers, such as studies evaluating population genetic structure (Veríssimo et al., 2010), spatial versus temporal population structure (Thorburn et al., 2018), assessment of genetic diversity levels and evolution (Gračan et al., 2020), and gene function and expression (Cutler et al., 2022). In addition, studies on reproductive biology, such as those by Lage et al. (2008) and Veríssimo *et al.* (2011) in the North Atlantic Ocean, detected polyandry using microsatellite markers.

Advancements in molecular techniques have enabled the use of genetics to study reproductive biology from a new perspective, allowing for accurate kinship assignment and detection of polyandry through multiple paternity by analyzing female and offspring DNA (Green et al., 2017; Liu et al., 2020; Nevatte et al., 2023). 

Single nucleotide polymorphisms (SNPs) are widely distributed genomic markers with the main advantage of being applicable to non-model organisms (Helyar et al., 2011; Peterson et al., 2012). They enable the detection of neutral and adaptive regions, which enhance the robustness of population genetic parameters and facilitates the identification of adaptive processes (Allendorf et al., 2010). These characteristics make SNPs an ideal marker type for conservation studies (Funk et al., 2012), population genetics, and ecology in various fish species (Zhang et al., 2015; Cruz et al., 2017; DiBattista et al., 2017; Colloca et al., 2020; Adachi *et al.,* 2023; Cruz *et al.,* 2023; Torres et al., 2024), as well as for studies of relatedness and multiple paternity (Flanagan and Jones, 2019; Bester-van der Merwe et al., 2019; Marie et al., 2019; Duchatelet et al., 2020; Liu et al., 2020; Nevatte et al., 2023). Genomic approaches, such as double-digest restriction site-associated DNA sequencing (ddRADseq), can provide informative SNPs (Peterson *et al.,* 2012) that reveal insights into the reproductive strategies employed by *S. acanthias*.

No populations of *S. acanthias* have previously been analyzed for genetic diversity and reproductive patterns with SNPs markers. Thus, the main goal of this study was to investigate the reproductive patterns of *S. acanthias*, in the Southwest of the Atlantic Ocean, testing the hypothesis of the maintenance of polyandry with multiple paternity in this species.

## Material and Methods

### Sample collection and DNA extraction

Muscle tissue was obtained from 40 samples of *Squalus acanthias* from six litters (mothers and their pups), collected from two locations in Argentina: Mar del Plata (MP = 17) in the north and Puerto de Santa Cruz (PSC = 23) in the south. The litter sizes ranged from 3 to 12 pups per litter. Tissues from all individuals were deposited in the fish collection of the Laboratory of Fish Biology and Genetics, UNESP, in Botucatu, São Paulo, Brazil. DNA samples were extracted from muscle tissue fragments preserved in 95% ethanol using the Wizard^®^ Genomic DNA Purification Kit (Promega, Madison, WI, USA), following the manufacturer’s solution-based DNA extraction protocol for animal tissues.

To confirm sample identification, the COI barcode region was amplified according to Hebert et al. (2003) for DNA barcode analyses of six samples, corresponding to the mothers of each litter. PCR amplicons were visualized on a 1% agarose gel and bi-directionally sequenced using the BigDye Terminator v.3.1 Cycle Sequencing Kit (Applied Biosystems, Inc.) on an ABI 3500 capillary sequencer, following the manufacturer’s instructions. The sequences were aligned in Geneious 4.8.5 (Kearse et al., 2012). Genetic distances were estimated using the Kimura-2-Parameter Model (K2P) (Kimura, 1980). The final matrix contained 26 sequences, including six obtained in the present study and 20 retrieved from the Barcode of Life Data System - BOLD (https://www.boldsystems.org/).

A maximum likelihood phylogenetic reconstruction was performed to construct a tree from the pairwise distances estimated using the General Time Reversible (GTR+I) substitution model. The best-fit substitution model was selected using the MEGA X model selection tool (Kumar et al., 2018). The tree was tested by bootstrap with 1,000 pseudoreplicates (Felsenstein, 1985), and all sequences analyzed were submitted to GenBank (Accession nos. PQ142937, PQ142938, PQ142939, PQ142940, PQ142941, PQ142942).

### SNP library construction

**Figure 2 -:**
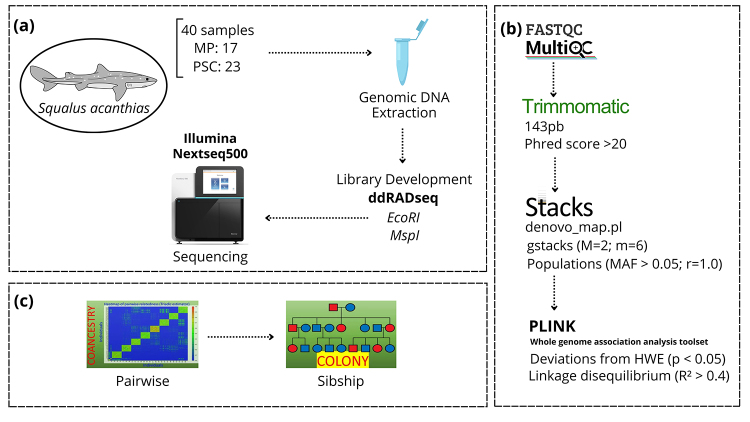
Workflow used for the ddRADseq technique (Double-digest restriction site-associated DNA sequencing): (a) Sampling locations and DNA sequencing. MP = Mar del Plata and PSC = Puerto de Santa Cruz. Genomic DNA was extracted and libraries were prepared using the restriction enzymes EcoRI and MspI. (b) Bioinformatic processing and filtering, including FastQC/MultiQC, TRIMMOMATIC, STACKS (de novo pipeline and parameters applied), and PLINK for filtering loci deviating from Hardy-Weinberg equilibrium (HWE) and in linkage disequilibrium. MAF = Minor Allele Frequency. (c) Kinship analyses, including pairwise relatedness estimation using COANCESTRY and sibship reconstruction using COLONY.

The 40 samples of *S. acanthias* were used for the ddRADseq technique (Peterson et al., 2012) (Figure 2). The *Eco*RI and *Msp*I restriction enzymes were used for digestion, according to the method described by Campos et al. (2017). Following the digestion, a pair of adaptors were attached to the fragments of each enzyme. The Nextera® Index Primers (Illumina, San Diego, CA, USA) i5 and i7 (Nextera DNA CD Indexes, 96 indexes, 96 samples) were used to index the samples. A pool of processed samples was prepared and submitted to 1% agarose gel electrophoresis. The fragments within 300-500 base pairs (bp) were removed and purified using the Wizard^®^ SV Gel and PCR Clean-Up System kit (Promega, Madison, WI, USA). The pool was then sequenced using an NGS Illumina Nextseq500 at the UNESP Biotechnology Institute (IBTEC), in Botucatu, São Paulo, Brazil.

After sequencing, the quality was assessed using FastQC (Andrews *et al*., 2015) and MultiQC (Ewels et al., 2016). All reads were trimmed to 143 bp using TRIMMOMATIC (Bolger, Lohse and Usadel, 2014) for the removal of adapters. All retained reads presented a Phred quality score above 20. The filtered and trimmed sequences were analyzed using Stacks 2.0 (Catchen et al., 2013), according to the protocol described by Rochette and Catchen (2017). As a reference genome was unavailable, the Stacks program *denovo_map.pl* was used to assemble the loci. The parameter optimization performed with the values (M=2, m=6, n=1) was in accordance with the recommendations provided by Paris, Stevens and Catchen (2017). The ‘*ustacks*’ unit was applied to build the stacks from the filtered reads. Subsequently, ‘*cstacks*’ was used to generate a reference catalogue. The ‘*sstacks*’ unit aligned reads from each sample with the catalogue, and the ‘*gstacks*’ unit called the variants. Finally, the Stacks *populations* pipeline was used with the application of two SNP filters: the first filter selected SNPs that occurred in 100% of individuals (r=1.0) per population, and the second filter excluded SNPs with an MAF (Minor Allele Frequency) value <0.05. The output files from Stacks were converted into other formats for input into the remaining programs using PGDSpider 2.1.1.5 (Lischer and Excoffier, 2011).

### Multiple paternity

For the assessment of multiple paternity, the different SNP dataset comprising the six litters, were subjected to PLINK 1.9 (Chang et al., 2015) for the application of quality control filters, such as the removal of samples based on the rate of missing data per individual, using mind = 0.1, which removes all samples with more than 10% missing genotypes. Finally, SNPs that deviated from the Hardy-Weinberg equilibrium were removed, and linkage disequilibrium filters were applied with the following parameters: window size = 40, step size = 5, and R² threshold = 0.4. 

The presence of multiple paternity in the litters was tested using two methods: pairwise relatedness and sibship analysis. Pairwise relatedness between mothers and pups were calculated using COANCESTRY (https://www.zsl.org/about-zsl/resources/software/coancestry; Wang, 2011). To determine which of the seven different estimators available in the program was most suitable for our data, we simulated 300 dyads for various relationship categories, including unrelated (r = 0), parent-offspring (r = 0.5), full-siblings (r = 0.5), and half-siblings (r = 0.25), based on the allele frequencies of the final filtered datasets for each litter of *Squalus acanthias*. Allele frequency information was filtered for each litter using R and the Related 1.0 package (https://github.com/timothyfrasier/related; Pew et al., 2015), an R implementation of COANCESTRY.

The simulation included a conservative genotyping error rate of 0.01 for each locus, with no missing data or allelic dropout. Pairwise relatedness was then calculated for the simulated dyads with the seven estimators, using the default value (100) for the number of reference individuals used for the triadic likelihood estimator (TrioML). The best relatedness estimator was identified based on both its accuracy (closeness to the true value) and precision (variation around the estimated values), as suggested by Attard et al. (2018). This involved examining Pearson’s correlation coefficient calculated for each estimator by COANCESTRY. Simulated dyad data for each relationship category were used to calculate means and standard deviations and to construct box plots for visual assessment of variance. The estimators used were Wang and LynchLi, which are modified to handle small sample sizes and provide unbiased estimates of relatedness (Wang, 2017). Therefore, these estimators were selected for the empirical analysis. The parameters for the empirical analysis were the same as those used in the simulation, with genotyping error accounted for in the analysis. The genotype input file containing all individuals was generated with Related, and all other required files were created following the instructions in the COANCESTRY manual.

To determine the most likely number of sires for each litter, a sibship analysis was conducted using COLONY (ver. 2.0.7.0, https://www.zsl.org/about-zsl/resources/software/colony; Jones and Wang, 2010). The program uses a full-likelihood approach to infer relationships among the offspring (full siblings, half siblings, or unrelated) and can reconstruct the genotypes of potential parents. For the analyses, COLONY assumes that the loci are in Hardy-Weinberg equilibrium (HWE) and linkage equilibrium.

The program uses a full-likelihood approach to infer relationships among the offspring (full siblings, half siblings, or unrelated) and can reconstruct the genotypes of potential parents. For the analyses, COLONY assumes that the loci are in HWE and linkage equilibrium. The genotypes of the pups and their mothers were included in the sibship analysis with COLONY, specifying this maternal relationship in the project file. Each litter was analyzed separately, using parameters similar to those described by Nevatte et al. (2023), as follows: males and females were set to a polygamous mating system, with no inbreeding and no clones present among the offspring. Considering that sharks are diploid and dioecious organisms, these options were selected. The full-likelihood method was chosen as the analysis method, with medium likelihood precision, medium run length, and five runs. The initial random number seed for the first run was set to 1234. No sibship prior was applied, sibship scaling was set to the default (Yes), and allele frequencies were not updated. The markers were set as codominant, with an allelic dropout rate of 0 and a genotyping error rate of 0.01. Allele frequencies were estimated during the analysis.

In the context of relatedness and sibship analysis performed by the COLONY software, inclusion and exclusion probabilities are metrics that evaluate the accuracy and reliability of paternity assignments and relationships among individuals. A high inclusion probability (above 0.99) indicates confidence in the data, showing that the individuals assigned as parents are indeed the biological parents of the offspring. This means that the COLONY results have high certainty and are reliable. A high exclusion probability (above 0.99) indicates strong confidence that any individual not identified as a parent is indeed not the biological parent of the offspring. This reinforces the accuracy of the assignments made, minimizing the possibility of significant errors.

High inclusion and exclusion probabilities are crucial to ensure that relatedness and sibship assignments are accurate. They provide the necessary level of certainty to infer biological relationships with confidence, which is fundamental in population genetics and evolutionary biology studies. When both inclusion and exclusion probabilities are greater than 0.99 within the same sample group, a high degree of confidence can be assigned to the results of the COLONY analysis. This indicates that the assigned parents are indeed biological parents, and that any other individual was correctly excluded as a possible parent. This significantly enhances the credibility of the results and conclusions derived from the genetic study. Therefore, to estimate the number of males contributing to each litter in our data, only Prob (Inc) and Prob (Excl) values greater than 99% were considered.

### Ethics permit and approval statement

All *Squalus acanthias* specimens analyzed in this study were obtained from targeted fishing operations in two localities along the Argentine coast. The samples were provided by collaborating researchers (Dr. Sergio M. Delpiani and Dr. Gabriela Delpiani, Investigaciones Marinas y Costeras, Argentina). Sampling was conducted in accordance with relevant local regulations; no specific permit number was issued.

## Results

The adult specimens used in this study were molecularly identified with DNA barcoding to confirm their previous morphological identification. The barcode sequences obtained ranged in length from 642 to 650 base pairs, revealing high percentages of similarity (>99%) with *S. acanthias* deposited in BOLD (Table S2). The intraspecific genetic distances values were 0.3%. The results of the Maximum Likelihood (ML) tree showed a single group for *S. acanthias*, formed by the adult individuals of this study, collected in two regions of Argentina, and the individuals extracted from the BOLD collected in different regions of the Atlantic Ocean (Figure S1). 

After confirming the specimen identification, a ddRAD library was developed for 40 samples of *S. acanthias* resulting in 94,308,943 raw data reads ranging from 1,082,597 to 3,124,731 reads per sample. After quality filtering, 51,582,038 reads were kept, ranging from 594,373 to 1,873,556 (Table S3). All reads were standardized with 143 bp, and after quality filtering, we obtained 1,021 SNPs (litter 1), 1,090 SNPs (litter 2), 1,124 SNPs (litter 3), 1,150 SNPs (litter 4), 1,620 SNPs (litter 5), and 1,480 SNPs (litter 6), which were used in subsequent analyses. 

### Multiple paternity

The pairwise relatedness and sibship analyses revealed the presence of multiple paternity in *S. acanthias*. The relatedness estimates using COANCESTRY for the Lynchli and Wang estimators were nearly identical for all litters and are reported here (Table 1). For litter 1 (8 pups), half-sibling relationships were found for 3 pups, with pups 104841, 104843 and 104846 being half-siblings pups (Lynchli range: 0.352-0.362 and Wang range: 0.372-0.375). For litter 2 (3 pups), half-sibling relationships were found for 2 pups, with pups 104850 and 104851 being half-siblings (Lynchli 0.329 and Wang 0.349). For litter 3 (3 pups), half-sibling relationships were found for 2 pups (Lynchli: 0.388 and Wang 0.406). For litter 4 (4 pups), half-sibling relationships were found for 2 pups, with pup 104859 being a half-sibling of pup 104860 (Lynchli 0.306 and Wang 0.321). For litter 5 (12 pups), half-sibling relationships were found for 5 pups, with pups 104862, 104863, 104864, 104865, and 104866 being half-siblings (Lynchli range: 0.065-0.229 and Wang range: 0.090-0.246). For litter 6 (4 pups), half-sibling relationships were found for all 4 pups (Lynchli range: 0.204-0.353 and Wang range: 0.227-0.373).

**Table 1 -:** Relatedness estimates generated by COANCESTRY for the six litters, using the Lynchli and Wang estimators.

Litter	Number of Pups	Half-Sibling Pups	Lynchli Range	Wang Range
1	8	104841, 104843, 104846	0.352-0.362	0.372-0.375
2	3	104850, 104851	0.329	0.349
3	3	104854, 104855	0.388	0.406
4	4	104859, 104860	0.306	0.321
5	12	104862, 104863, 104864, 104865, 104866	0.065-0.229	0.090-0.246
6	4	All	0.204-0.353	0.227-0.373

The sibship analysis with COLONY confirmed multiple paternity in the six litters analyzed (Table 2). In litter 1, the analysis indicated that five males sired the litter. Father 1 would have sired three pups (104840, 104844, and 104846) with low Inc and Exc probabilities (0.58). Father 2 would have sired two pups (104841 and 104847) with Inc and Exc probabilities (>0.99). Father 3 would have sired one pup (104842) with a high Inc probability (>0.99) and low Exc probability (0.31). Father 4 would have sired one pup (104843) with Inc and Exc probabilities (>0.99). Father 5 would have sired one pup (104845) with a high Inc probability (>0.99) and low Exc probability (0.47). Thus, considering only inclusion and exclusion probabilities (>0.99), this litter had at least two distinct fathers: Father 2 with two pups and Father 4 with one pup.

**Table 2 -:** Results from the sibship analysis performed using COLONY for the litters of *Squalus acanthias*. Only probabilities (>99%) are reported. The inclusion probability Prob (Inc.), exclusion probability Prob (Exc.), and vouchers of the pups for each full-sibling family are presented.

Litter	Full-sibling family/Inferred father	Prob (Inc.)	Prob (Exc.)	Pups
1	F2	1.000	1.000	104841, 104847
	F4	1.000	1.000	104843
3	F1	1.000	1.000	104853
	F2	1.000	1.000	104854
	F3	1.000	1.000	104855
4	F1	1.000	1.000	104857
	F2	1.000	1.000	104858
	F3	1.000	1.000	104859
	F4	1.000	1.000	104860
5	F1	1.000	1.000	104862, 104863,104864
	F2	1.000	1.000	1104865, 104866, 104867, 104868
	F3	1.000	1.000	104869, 104870, 104871, 104872, 104873
6	F1	1.000	1.000	104875
	F2	1.000	1.000	104876
	F3	1.000	1.000	104877
	F4	1.000	1.000	104878

Additionally, the pups identified here as half-siblings (104841 and 104843), sired by fathers 2 and 4 respectively, were consistent with the results found in the pairwise relatedness estimates conducted with COANCESTRY. In litter 2 (3 pups), the analysis indicated that the litter was sired by two males. Father 1 would have sired two pups (104849 and 104850), but the Inc and Exc probabilities were low (0.13), and Father 2 would have sired only one pup (104851), with Inc and Exc probabilities (>0.99). 

The half-sibling relationship between 104850 and 104851 was also confirmed by the pairwise analysis conducted with COANCESTRY. In litter 3 (3 pups), the analysis indicated that the litter was sired by three males, meaning that each pup had a different father. Father 1 would have sired one pup (104853), Father 2 one pup (104854), and Father 3 one pup (104855). All with Inc and Exc probabilities (>0.99). The half-sibling relationship between 104854 and 104855 was also confirmed by the pairwise analysis conducted by COANCESTRY. In litter 4 (4 pups), the analysis indicated that the litter was sired by four males, with each pup having a different father. Father 1 sired one pup (104857), Father 2 one pup (104858), Father 3 one pup (104859), and Father 4 one pup (104860). All with Inc and Exc probabilities (>0.99). The half-sibling relationship between pups 104859 and 104860 was confirmed by the pairwise analysis conducted by COANCESTRY. 

In litter 5, the largest litter in this study (12 pups), the analysis indicated that the litter was sired by three males. Father 1 would have sired three pups (104862, 104863, and 104864). Father 2 would have sired four pups (104865, 104866, 104867, and 104868). Father 3 would have sired five pups (104869, 104870, 104871, 104872, and 104873). All with Inc and Exc probabilities (>0.99). The half-sibling relationship between 104862, 104863, 104864, 104865, and 104866 and the other pups was confirmed by the pairwise analysis conducted by COANCESTRY. Finally, in litter 6 (4 pups), the analysis indicated that the litter was sired by four males, with each pup having a different father. Father 1 sired one pup (104875), Father 2 one pup (104876), Father 3 one pup (104877), and Father 4 one pup (104878). All with Inc and Exc probabilities (>0.99). The half-sibling relationship between 104875 and 104876 and the other pups was confirmed by the pairwise analysis conducted by COANCESTRY.

## Discussion

The results of this study offer insights into the reproductive strategies of *Squalus acanthias* along the Argentine coast, particularly regarding the occurrence of polyandry and multiple paternity, which have not been previously evaluated using SNP markers. The application of the ddRADseq methodology enabled a detailed analysis of the species’ genome, providing a robust basis for identifying SNP markers and inferring relationships among embryos. Additionally, this study extends the geographic range of documented multiple paternity occurrences. 

In terms of fecundity, moderate levels were observed, with litter sizes ranging from three to twelve offspring. This range is aligned with the established reproductive parameters of the species, which range from one to twenty-one offspring per gestation cycle (Compagno, 1984). Conversely, congeneric species such as *Squalus megalops* (Macleay, 1881) typically produce two to four pups per pregnancy (Watson and Smale, 1998), whereas *Squalus blainvillei* (Risso, 1827) typically produces an average of three to four offspring per litter (Ebert et al., 2013).

Comparing our findings with previous studies based on microsatellites, such as Lage et al. (2008), our results reveal a substantial improvement in the detection of multiple paternity. While Lage *et al.* reported multiple paternity predominantly in larger litters (>5 pups), we detected multiple paternity in both small litters (as few as 3 offspring) and in larger litters (9 and 12 offspring). This demonstrates that the commonly accepted association between litter size and the probability of multiple paternity may have been partially driven by methodological limitations rather than by biological constraints.

The use of thousands of SNP markers greatly increased the resolution of our kinship analyses, allowing for more accurate estimates of the number of sires and a clearer distinction among relatedness categories within each litter. The high SNP recovery per litter provided more informative data than traditional microsatellite panels, enabling us to uncover patterns of paternity that were likely undetectable in previous studies. Additionally, our findings are consistent with Colonello et al. (2016), who showed that female reproductive cycles in *Squalus acanthias* exhibit variable fertility and may experience increased embryo loss during late gestation due to fishing-related stress.

Taken together, our results contribute new evidence that multiple paternity occurs even in small litters and highlight the importance of high-resolution genomic markers for accurately describing mating systems in elasmobranchs. These findings provide a foundation for further discussion of reproductive strategies in *S. acanthias*.

According to Veríssimo et al. (2011), who analyzed 29 litters of *S. acanthias* (with five to seven pups per litter) using seven microsatellite markers, the study revealed that litter size increased with female size but was similar between polyandrous and monogamous females. This suggests that, although litter size may be influenced by female size, multiple paternity did not have a significant impact on litter size, as also observed in our study. Thus, polyandry in *S. acanthias* may be influenced by other ecological or behavioral factors, rather than just litter size and fecundity.

The evidence found in this study of multiple paternity occurring in both small and large litters, coupled with the high number of males (fathers) contributing to the fertilization of smaller litters, could indicate that this reproductive strategy is being utilized by the species to help maintain genetic variability. This phenomenon could be a response to a decrease in available females for mating, potentially explaining why a litter of 3 to 4 pups may have one father for each pup. Literature reports also indicate that when a female mates with multiple males during the same breeding season (polyandry), the first male to copulate tends to father the largest number of pups in the litter (Orr and Brennan, 2015). Thus, when multiple pups are attributed to different fathers, it may be possible to identify the first male to mate with the female.

A recent study by Andrade et al. (2024) confirms that *S. acanthias* is more frequently captured in shallow coastal areas (between 19 and 150 meters deep), with females being more abundant and larger than males. Some females were found pregnant with advanced-stage embryos, although the embryos were aborted before sampling. This further supports the observed size and sex segregation relative to depth in this species. Understanding bathymetric variation is crucial for managing and conserving fishery resources. Additionally, *S. acanthias* exhibits sexual dimorphism, with females being larger than males and taking longer to reach sexual maturity (Colonello et al., 2016). This species also demonstrates sex and size segregation, known as bathymetric variation, with males often found in deeper waters and females in shallower depths (Andrade *et al.,* 2024). This segregation increases the vulnerability of females to population declines caused by intensive fishing (Shepherd et al., 2002; Lamarca et al., 2020b). 

Throughout the Atlantic Ocean, *S. acanthias* populations have been affected by varying levels of fishing pressure (Colonello et al., 2016). In the southwestern Atlantic, this species is often caught as bycatch by major industrial fisheries and discarded at sea (Massa et al., 2004; Cedrola et al., 2012; Góngora et al., 2023). In the North Atlantic, along the coasts of the USA and Canada, this shark has been significantly impacted by continuous removal since the early 20th century (Rago et al., 1998; Wallace et al., 2009; Dell’Apa et al., 2015). This exploitation can decrease reproduction rates due to reduced abundance or altered sex ratios of mature individuals (Daly-Engel et al., 2006). Capturing more females can significantly reduce the species’ chances of survival and persistence. 

Although multiple paternity may not be directly related to litter size, female size is crucial for reproductive capacity and the production of more offspring. Therefore, protecting these females in their natural habitat is essential for species survival. Given that *S. acanthias* has one of the longest gestation periods among elasmobranchs and that larger females may produce more pups, protecting this species is crucial for ensuring that both males and females reach sexual maturity and reproduce, thereby ensuring the species’ survival.

Many sharks and rays have already become extinct, with about a third of species lost due to human pressures, and fishing has been responsible for 67.3% of these extinctions (Dulvy et al., 2021). Maintaining genetic variability is vital for species survival. The findings of this study emphasize the need for management policies that consider genetic diversity and reproductive practices, particularly for species with complex life histories and low stock replacement rates. Multiple paternity can help maintain gene flow and genetic diversity (Newcomer et al., 1999; Hoekert et al., 2002; Hudson, 2022) and may aid in reducing infertility (Schmidt et al., 2010; Hudson, 2022). Therefore, multiple matings and the occurrence of multiple paternity can be essential for supporting genetic diversity within the species.

The capture of pregnant females, which often abort due to stress, represents a significant challenge. Future research should focus on expanding sampling and applying advanced genomic techniques to explore other aspects of the reproductive biology of *S. acanthias*. Studies in different geographical regions and ecological contexts would also be valuable for understanding variations in reproductive strategies. This study enhances our understanding of the complex reproductive strategies of *S. acanthias*, highlighting the prevalence of multiple paternity and the need for conservation measures that account for the genetic diversity and reproductive practices of the species along the Argentine coast.

## Conclusion

In summary, we provide evidence that multiple paternity continues to be a reproductive strategy used by *Squalus acanthias*. This was the first study conducted with SNPs, and the markers showed great efficiency in detecting multiple paternity in both large and small litters. Our findings on multiple paternity are consistent with those already observed in litters of various elasmobranch species and highlight that the occurrence of this behavior in both large and small litters may be related to intrinsic factors of the group. Therefore, this study demonstrates the importance of the persistence of multiple paternity, especially in groups more vulnerable to fishing pressure, contributing to the maintenance of genetic diversity and species persistence.

## Supplementary material

Figure S1 - Maximum Likelihood tree of *Squalus acanthias* specimens based on mitochondrial cytochrome c oxidase subunit I (COI) gene sequences under the K2P model.

Table S1 - Orders, families, genera, and species of viviparous and oviparous elasmobranchs investigated in studies on the occurrence of multiple paternity. Numbers preceding the references indicate the occurrence of polyandry in the corresponding species.

Table S2 - Identification of the samples used in the study, highlighting the location of the specimens from this study marked in gray (●).

Table S3 - Summary of the sequencing and processing of 40 samples of *Squalus acanthias*.

## Data Availability

Accession numbers for genetic sequences generated for this study are provided in Table S2 and in the Methods section of the manuscript.
