# Supplementary material for: Evaluation of the occurrence of multiple paternity in Squalus acanthias in the South Atlantic region using nuclear markers
Source: Genet Mol Biol. 2026 Jul 3;49(2):e20260013. doi: 10.1590/1678-4685-GMB-2026-0013 (PMC13331067; doi:10.1590/1678-4685-GMB-2026-0013)
Supplement: Figure S1 - [file 1415-4757-GMB-49-2-e20260013-s1.pdf]

Genetics and Molecular Biology

https://doi.org/10.1590/1678-4685-GMB-2026-0013

Supplementary Material to: Evaluation of the occurrence of multiple paternity in *Squalus acanthias* in the South Atlantic region using nuclear markers

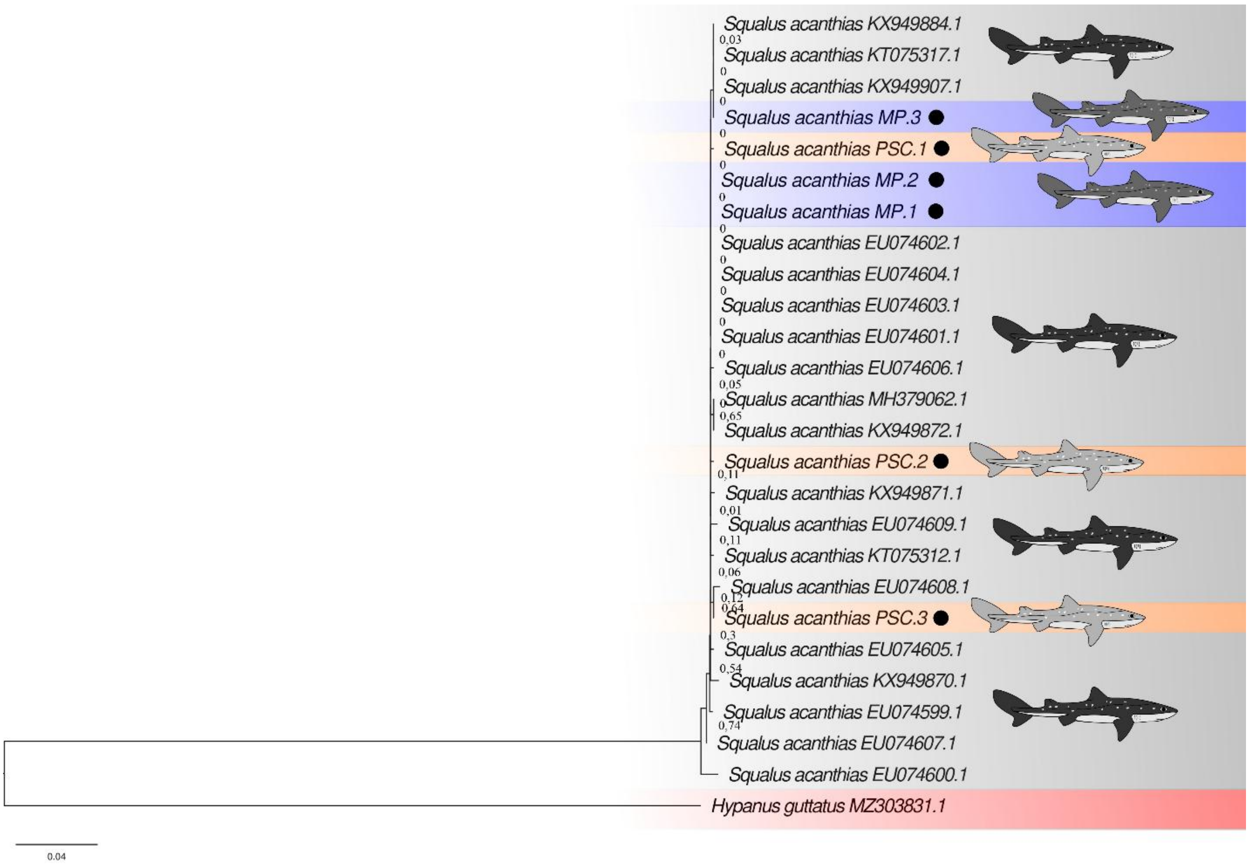

Figure S1 - Maximum Likelihood tree of *Squalus acanthias* specimens based on mitochondrial cytochrome c oxidase subunit I (COI) gene sequences under the K2P model. The asterisks (*) indicate the six samples sequenced in this study. Sequences highlighted in gray correspond to reference sequences obtained from public databases. Sequences in blue represent *S. acanthias* samples from Mar del Plata (MP) generated in this study, while those in orange represent *S. acanthias* samples from Puerto de Santa Cruz (PSC) generated in this study. The sequence highlighted in red corresponds to *Hypanus guttatus*, used as outgroup.
