# Supplementary material for: Evaluation of the occurrence of multiple paternity in Squalus acanthias in the South Atlantic region using nuclear markers
Source: Genet Mol Biol. 2026 Jul 3;49(2):e20260013. doi: 10.1590/1678-4685-GMB-2026-0013 (PMC13331067; doi:10.1590/1678-4685-GMB-2026-0013)
Supplement: Table S1 - [file 1415-4757-GMB-49-2-e20260013-s2.pdf]

Genetics and Molecular Biology
https://doi.org/10.1590/1678-4685-GMB-2026-0013

Supplementary Material to: Evaluation of the occurrence of multiple paternity in *Squalus acanthias* in the South Atlantic region using nuclear markers

Table S1 - Orders, families, genera, and species of viviparous and oviparous elasmobranchs investigated in studies on the occurrence of multiple paternity. Numbers preceding the references indicate the occurrence of polyandry in the corresponding species.

Order	Family	Occurence of polyandry[1]	Popular name	Reproduction type	References
Carcharhiniformes	Carcharhinidae	*Carcharhinus acronotus* (Poey, 1860)	Blacknose shark	Placental viviparity	1. Barker et al., (2019)
		Carcharhinus leucas (Valenciennes, 1839)	Bull shark	Yolk-only viviparity	1. Pirog et al., (2015); Pirog et al., (2019)
		Carcharhinus amblyrhynchos (Bleeker, 1856)	Grey reef shark	Placental viviparity	1. Green et al., (2017)
		Carcharhinus obscurus (Lesueur, 1818)	Dusky shark	Yolk-only viviparity	1. Rossouw et al., (2016)

Order	Family	Occurence of polyandry[1]	Popular name	Reproduction type	References
		Carcharhinus plumbeus (Nardo, 1827)	Sandbar shark	Placental viviparity	1. Daly-Engel et al., (2006); Portnoy et al., (2007); Daly-Engel et al., (2007); Marino et al., (2015)
		Carcharhinus altimus (Springer, 1950)	Bignose shark	Placental viviparity	1. Daly-Engel et al., (2006)
		Carcharhinus isodon (Valenciennes, 1839)	Finetooth shark	Placental viviparity	1. Nash et al., (2021)
		Negaprion acutidens (Rüppell, 1837)	Sicklefin lemon shark	Placental viviparity	1. Mourier et al., (2013)
		Negaprion brevirostris (Poey, 1868)	Lemon shark	Placental viviparity	1. Feldheim et al., (2001); Feldheim et al., (2004); DiBattista et al., (2008)
		Prionace glauca (Linnaeus, 1758)	Blue shark	Placental viviparity	1. Armada-Tapia et al., (2022)
	Scyliorhinidae	*Scyliorhinus canicula* (Linnaeus, 1758)	Smallspotted catshark	Egg-laying	1. Griffiths et al., (2011)
	Sphyrnidae	*Sphyrna lewini* (Griffith & Smith, 1834)	Scalloped hammerhead	Placental viviparity	1. Rossouw et al., (2016); Green et al., (2017); Marie et al., (2019)
		Sphyrna tiburo (Linnaeus, 1758)	Bonnethead	Placental viviparity	1. Chapman et al., (2004)

Order	Family	Occurence of polyandry[1]	Popular name	Reproduction type	References
		Mustelus mustelus (Linnaeus, 1758)	Smooth-hound shark	Placental viviparity	1. Marino et al., (2015a); Rossouw et al., (2016)
		Mustelus punctulatus Risso, 1827	Blackspotted smooth-hound	Yolk-only viviparity	1. Marino et al., (2015)
		Mustelus henlei (Gill, 1863)	Brown smooth-hound shark	Placental viviparity	1. Byrne & Avise (2012); Chabot & Haggin (2014); Rendón-Herrera et al., (2022)
		Mustelus asterias Cloquet, 1819	Starry smooth-hound	Yolk-only viviparity	1. Farrell et al., (2014)
	Triakidae	*Mustelus antarcticus* Günther, 1870	Gummy shark	Yolk-only viviparity	1. Boomer et al., (2013)
		Mustelus lenticulatus Phillipps, 1932	Spotted estuary smooth-hound	Yolk-only viviparity	1. Boomer et al., (2013)
		Galeorhinus galeus (Linnaeus, 1758)	Tope shark	Yolk-only viviparity	1. Hernandez et al., (2014)
		Triakis semifasciata Girard, 1855	Leopard shark	Yolk-only viviparity	1. Nosal et al., (2013)
Hexanchiformes	Hexanchidae	*Hexanchus griséus* (Bonnaterre, 1788)	Bluntnose sixgill shark	Yolk-only viviparity	1. Larson et al., (2011)
Lamniformes	Lamnidae	*Isurus oxyrinchus* Rafinesque, 1810	Shortfin mako	Viviparity with oophagy	1. Corrigan et al., (2015); Liu et al., (2020)

Order	Family	Occurence of polyandry[1]	Popular name	Reproduction type	References
	Carchariidae	*Carcharias taurus* Rafinesque, 1810	Sand tiger shark	Viviparity with oophagy	1. Chapman et al., (2013); Townsend et al., (2015)
Orectolobiformes	Ginglymostomatidae	*Ginglymostoma cirratum* (Bonnaterre, 1788)	Nurse shark	Yolk-only viviparity	1. Saville et al., (2002); Heist et al., (2011);
Squaliformes	Squalidae	*Squalus albicaudus* Viana, Carvalho & Gomes, 2016	Brazilian whitetail dogfish	Yolk-only viviparity	1. Lamarca et al., (2020)
		Squalus mitsukurii Jordan & Snyder, 1903	Shortspine spurdog	Yolk-only viviparity	1. Daly-Engel et al., (2010)
		Squalus acanthias Linnaeus, 1758	Spiny-dogfish	Yolk-only viviparity	1. Lage et al., (2008); Veríssimo et al., (2011); Craven et al., (2018); This study
Pristiophoriformes	Pristiophoridae	*Pristiophorus cirratus* (Latham, 1794)	Australian sawsharks	Yolk-only viviparity	1. Nevatte et al., (2023)
Rajiformes	Rajidae	*Raja clavata* Linnaeus, 1758	Thornback ray	Egg-laying	1. Chevolot et al., (2007)
Myliobatiformes	Myliobatidae	*Aetobatus narinari* (Euphrasen, 1790)	Whitespotted eagle ray	Viviparity with uterine milk	1. Janse et al., (2013)

Order	Family	Occurence of polyandry[1]	Popular name	Reproduction type	References
	Urotrygonidae	*Urobatis halleri* (Cooper, 1863)	Round stingray	Viviparity with uterine milk	1. Lyons et al., (2017)
	Potamotrygonidae	*Potamotrygon Leopoldi* Castex & Castello, 1970	Xingu freshwater stingray	Viviparity with uterine milk	1. Torres et al., (2022)
