# Supplementary material for: Evaluation of the occurrence of multiple paternity in Squalus acanthias in the South Atlantic region using nuclear markers
Source: Genet Mol Biol. 2026 Jul 3;49(2):e20260013. doi: 10.1590/1678-4685-GMB-2026-0013 (PMC13331067; doi:10.1590/1678-4685-GMB-2026-0013)
Supplement: Table S2 - [file 1415-4757-GMB-49-2-e20260013-s3.pdf]

Genetics and Molecular Biology
https://doi.org/10.1590/1678-4685-GMB-2026-0013

Supplementary Material to: Evaluation of the occurrence of multiple paternity in *Squalus acanthias* in the South Atlantic region using nuclear markers

Table S2 - Identification of the samples used in the study, highlighting the location of the specimens from this study marked in gray (●). The sequences available in the online databases GenBank and BOLD systems are presented with their respective accession numbers and references.

Species	Location	GenBank acession	Sequence ID BOLD	Reference
Squalus acanthias ●	Argentina: Mar del Plata	PQ142940.1	-	Present study
Squalus acanthias ●	Argentina: Mar del Plata	PQ142941.1	-	Present study
Squalus acanthias ●	Argentina: Mar del Plata	PQ142942.1	-	Present study
Squalus acanthias ●	Argentina: Puerto de Santa Cruz	PQ142937.1	-	Present study
Squalus acanthias ●	Argentina: Puerto de Santa Cruz	PQ142938.1	-	Present study
Squalus acanthias ●	Argentina: Puerto de Santa Cruz	PQ142939.1	-	Present study
Squalus acanthias	Argentina	EU074609.1	FARG199-06	Mabragaña et al., 2011
Squalus acanthias	Argentina	EU074607.1	FARG201-06	Mabragaña et al., 2011
Squalus acanthias	Argentina	EU074601.1	FARG202-06	Mabragaña et al., 2011
Squalus acanthias	Argentina	EU074605.1	FARG203-06	Mabragaña et al., 2011
Squalus acanthias	Argentina	EU074606.1	FARG204-06	Mabragaña et al., 2011
Squalus acanthias	Argentina	EU074608.1	FARG205-06	Mabragaña et al., 2011
Squalus acanthias	Argentina	EU074603.1	FARG227-06	Mabragaña et al., 2011
Squalus acanthias	Argentina	EU074604.1	FARG228-06	Mabragaña et al., 2011
Squalus acanthias	Argentina	EU074600.1	FARG254-06	Mabragaña et al., 2011
Squalus acanthias	Argentina	EU074599.1	FARG255-06	Mabragaña et al., 2011
Squalus acanthias	Uruguay	EU074602.1	FARG334-07	Mabragaña et al., 2011

Species	Location	GenBank acession	Sequence ID BOLD	Reference
Squalus acanthias	United States of America	KT075312.1	ANGBF12855-15	Unpublished
Squalus acanthias	United States of America	KT075317.1	ANGBF12856-15	Unpublished
Squalus acanthias	United States of America: New Jersey	MH379062.1	ANGBF47276-19	Stoeckle et al., 2018
Squalus acanthias	France	KX949870.1	ANGBF13754-18	Verissimo et al., 2016
Squalus acanthias	France	KX949884.1	ANGBF13765-18	Verissimo et al., 2016
Squalus acanthias	South Africa	KX949907.1	ANGBF13785-18	Verissimo et al., 2016
Squalus acanthias	France	KX949871.1	ANGBF47273-19	Verissimo et al., 2016
Squalus acanthias	Portugal	KX949872.1	ANGBF47275-19	Verissimo et al., 2016
Hypanus guttatus	Brazil: Amazon costal	MZ303831.1	ARR65F138	Martins et al., 2021
