# Supplementary material for: Evaluation of the occurrence of multiple paternity in Squalus acanthias in the South Atlantic region using nuclear markers
Source: Genet Mol Biol. 2026 Jul 3;49(2):e20260013. doi: 10.1590/1678-4685-GMB-2026-0013 (PMC13331067; doi:10.1590/1678-4685-GMB-2026-0013)
Supplement: Table S3 - [file 1415-4757-GMB-49-2-e20260013-s4.pdf]

Genetics and Molecular Biology
DOI: https://doi.org/10.1590/1678-4685-GMB-2026-0013

Supplementary Material to: Evaluation of the occurrence of multiple paternity in *Squalus acanthias* in the South Atlantic region using nuclear markers

Table S3 - Summary of 40 samples sequencing and processing of *Squalus acanthias*. Asterisks* represent samples that were not sequenced in DNA barcode.

N	Sample ID	Litter	Locality			DNA barcode	NGS Raw Data	Final dataset
			City	Province of Argentina	Coast region			
1	104839	1	Mar del Plata	Buenos Aires	Central-eastern	yes	2.409.454	1.131.669
2	104840	1	Mar del Plata	Buenos Aires	Central-eastern	*	2.225.154	1.107.996
3	104841	1	Mar del Plata	Buenos Aires	Central-eastern	*	2.595.289	1.236.358
4	104842	1	Mar del Plata	Buenos Aires	Central-eastern	*	3.447.616	1.701.036
5	104843	1	Mar del Plata	Buenos Aires	Central-eastern	*	2.661.341	1.345.300
6	104844	1	Mar del Plata	Buenos Aires	Central-eastern	*	1.966.165	1.071.874
7	104845	1	Mar del Plata	Buenos Aires	Central-eastern	*	2.247.754	1.295.068
8	104846	1	Mar del Plata	Buenos Aires	Central-eastern	*	1.761.139	1.140.035
9	104847	1	Mar del Plata	Buenos Aires	Central-eastern	*	2.722.878	1.264.730
10	104848	2	Mar del Plata	Buenos Aires	Central-eastern	yes	2.635.520	1.277.589
11	104849	2	Mar del Plata	Buenos Aires	Central-eastern	*	2.215.224	1.135.566
12	104850	2	Mar del Plata	Buenos Aires	Central-eastern	*	2.279.136	1.087.173
13	104851	2	Mar del Plata	Buenos Aires	Central-eastern	*	1.327.398	656.813
14	104852	3	Mar del Plata	Buenos Aires	Central-eastern	yes	2.140.982	1.066.252
15	104853	3	Mar del Plata	Buenos Aires	Central-eastern	*	1.714.486	808.349
16	104854	3	Mar del Plata	Buenos Aires	Central-eastern	*	2.297.156	1.180.004
17	104855	3	Mar del Plata	Buenos Aires	Central-eastern	*	2.304.334	1.256.529
18	104856	4	Puerto de Santa Cruz	Santa Cruz	South	yes	1.817.923	859.345

N	Sample ID	Litter	Locality			DNA barcode	NGS Raw Data	Final dataset
			City	Province of Argentina	Coast region			
19	104857	4	Puerto de Santa Cruz	Santa Cruz	South	*	1.979.518	964.436
20	104858	4	Puerto de Santa Cruz	Santa Cruz	South	*	2.131.274	887.470
21	104859	4	Puerto de Santa Cruz	Santa Cruz	South	*	2.600.901	1.146.768
22	104860	4	Puerto de Santa Cruz	Santa Cruz	South	*	3.006.625	1.214.970
23	104861	5	Puerto de Santa Cruz	Santa Cruz	South	yes	2.420.747	1.174.841
24	104862	5	Puerto de Santa Cruz	Santa Cruz	South	*	1.082.597	541.427
25	104863	5	Puerto de Santa Cruz	Santa Cruz	South	*	2.547.519	1.335.102
26	104864	5	Puerto de Santa Cruz	Santa Cruz	South	*	2.560.784	1.156.564
27	104865	5	Puerto de Santa Cruz	Santa Cruz	South	*	2.640.081	1.309.306
28	104866	5	Puerto de Santa Cruz	Santa Cruz	South	*	2.572.571	1.151.486
29	104867	5	Puerto de Santa Cruz	Santa Cruz	South	*	2.678.957	1.427.219
30	104868	5	Puerto de Santa Cruz	Santa Cruz	South	*	2.093.257	1.365.734
31	104869	5	Puerto de Santa Cruz	Santa Cruz	South	*	2.400.524	1.033.641
32	104870	5	Puerto de Santa Cruz	Santa Cruz	South	*	2.180.316	1.159.222
33	104871	5	Puerto de Santa Cruz	Santa Cruz	South	*	2.727.283	1.446.120
34	104872	5	Puerto de Santa Cruz	Santa Cruz	South	*	2.527.244	1.418.287
35	104873	5	Puerto de Santa Cruz	Santa Cruz	South	*	2.439.703	1.079.665
36	104874	6	Puerto de Santa Cruz	Santa Cruz	South	yes	3.124.731	1.585.072
37	104875	6	Puerto de Santa Cruz	Santa Cruz	South	*	2.978.525	1.392.157
38	104876	6	Puerto de Santa Cruz	Santa Cruz	South	*	2.740.117	1.429.175
39	104877	6	Puerto de Santa Cruz	Santa Cruz	South	*	2.534.038	1.246.671
40	104878	6	Puerto de Santa Cruz	Santa Cruz	South	*	1.572.685	838.038
